# Is immunohistochemistry more sensitive than hematoxylin-eosin staining for identifying perineural or lymphovascular invasion in oral squamous cell carcinoma? A systematic review and meta-analysis

**DOI:** 10.4317/medoral.25114

**Published:** 2022-04-14

**Authors:** Ana Paula Negreiros Nunes Alves, Dayrine Silveira de Paula, Lia Vila Real Lima, Thinali Sousa Dantas, Mário Rogério Lima Mota, Fabrício Bitu Sousa, Paulo Goberlânio de Barros Silva

**Affiliations:** 1 Post-graduate program in Clinic Dentistry, Federal University of Ceará, Fortaleza, Brazil; 2 Post-graduate program in Dental Sciences, Unichristus, Fortaleza, Brazil

## Abstract

**Background:**

This study aimed to analyze whether immunohistochemistry (IHC) is more sensitive than hematoxylin-eosin (H&E) staining for identifying perineural invasion (PNI) or lymphovascular invasion (LVI) in oral squamous cell carcinoma (OSCC).

**Material and Methods:**

In this systematic review and meta-analysis (Prospective Register of Systematic Reviews – CRD 42021256515), data were obtained from six databases (PubMed, Scopus, LILACS, Web of Science, EBSCO, LIVIVO, Embase) and the grey literature. Cross-sectional observational studies of the diagnostic sensitivity of IHC for PNI and LVI were included. Studies were selected in two phases: first collection and reference retrieval. The Quality Assessment of Diagnostic Accuracy Studies-2 tool assessed study quality, while the Grading of Recommendations, Assessment, Development, and Evaluation (GRADE) approach assessed evidence quality. The meta-analysis (random effects model) was performed using MedCalc 18.2.1 software (MedCalc®) (*p*<0.05).

**Results:**

Four studies (560 patients with 295 biopsies) were analyzed. The combined sensitivity was 76% (95% confidence interval [CI], 44.30–97.19%) and specificity was 42% (95% CI, 23.40–62.02%). The positive predictive value (PPV) and negative predictive value (NPV) were 61% (95% CI, 49.78–71.53%) and 70% (95% CI, 37.63–94.43%). The overall accuracy was 58% (95% CI, 45.17–70.65%). The risk of bias was low, and GRADE analysis showed a very low certainty of evidence.

**Conclusions:**

Our data suggest that IHC staining to highlight PNI/LVI may be useful in cases in which H&E analysis results in a negative decrease in the prevalence of false-negative cases and underestimated treatment.

** Key words:**Mouth neoplasms, neoplasm invasiveness, blood vessels, peripheral nerves.

## Introduction

Oral squamous cell carcinoma (OSCC), the most common oral cancer, is defined as an invasive epithelial neoplasm with varying degrees of squamous differentiation and a propensity for early and extensive lymphatic metastasis (1,2).

One of the primary aspects of the diagnosis and prognosis of patients with OSCC is the knowledge of the histopathological characteristics of the lesion, such as perineural invasion (PNI) and lymphovascular invasion (LVI), which are recognized as indicators of locoregional recurrence, metastasis, and overall survival and a significant negative predictor of outcomes (3). However, there is much variation in the frequency of detection of these histological findings, which may contribute to understaging and consequent underestimation during therapeutic planning (4).

The histological findings of both invasions arise from subjective variables such as the number of blocks selected in the macroscopic examination and the care with which features are sought. The criteria applied by a pathologist during microscopic evaluation and objective variables such as tumor site and stage also contribute to discrepant evaluations. Furthermore, diagnosis has been established using conventional hematoxylin-eosin (H&E) staining, the gold standard owing to its low cost and easy handling (5).

Some immunohistochemical (IHC) markers have been used to identify vessels and nerves more precisely, contributing to the diagnosis of PNI and LVI in different diseases, including OSCC, in an attempt to overcome the limitations of conventional staining (6). As it results in a more detailed morphological analysis of structures related to their dissemination, IHC contributes significantly to better treatment guidance and prognosis establishment for patients (4,7,8).

The most significant limitation of IHC is its cost. Despite facilitating the diagnosis, algorithms should be developed to improve its cost-benefit for diagnosing PNI and LVI in OSCC. Thus, knowing that this technique can mitigate the underdiagnosis of these histological patterns that strongly impact prognosis, this systematic review aimed to evaluate whether IHC techniques have equal predictive value for PNI or LVI in patients with OSCC.

- Abbreviations

DSP – Dayrine Silveira de Paula; LVRL – Lia Vila Real Lima; PGBS – Paulo Goberlânio de Barros Silva; APNNA – Ana Paula Negreiros Nunes Alves; PNI – Perineural Invasion; LVI - Lymph Vascular Invasion; OSCC - Oral Squamous Cell Carcinoma.

## Material and Methods

- Protocol and Registration

The Preferred Reporting Items for Systematic Reviews and Meta-Analyses (PRISMA) (9) were followed to guide our study protocol. The study was registered with the International Prospective Register of Systematic Reviews (CRD 42021256515).

- Information Search and Search Strategy

A systematic review was conducted to answer the following question: “Does IHC analysis increase the sensitivity for diagnosing PNI and LVI in patients with oral cancer?” designed using the PECOS strategy as follows:

1. Population (P): Mouth cancer patients

2. Exposition (E): Use of IHC techniques

3. Control (C): Compared to conventional H&E

4. Outcome (O): Increases sensitivity for the diagnosis of PNI and LVI

5. Study design (S): cross-sectional, cohort, and case-control studies

Searches in each electronic database were performed using combinations of specific terms. Supplementary data for all search strategies are presented in the text (Supplement 1). Duplicate references were excluded using Rayyan® software.

- Inclusion Criteria

This systematic review included observational studies that evaluated PNI and LVI by at least conventional histological evaluation and an IHC marker to detect invasion.

- Exclusion Criteria

Studies that met the following exclusion criteria were not included in this investigation: 1) literature reviews; 2) case reports; 3) letters to the editor; 4) clinical observations; 5) articles describing particular opinions of specific authors; 6) book chapters; 7) meeting abstracts; and 8) studies evaluating predictive markers of tumor prognosis.

- Information Sources

Using appropriate search strategies, data were obtained from six major databases (PubMed, Scopus, LILACS, Web of Science, Embase, LiVivo, and EBSCOhost). Google Scholar, OpenGrey, and ProQuest were included as grey literature sources. The search was conducted without time restrictions and included all articles published in the databases on or before June 06, 2021. Appropriate truncations and word combinations were selected and adapted for each search. Additional information regarding the search strategies is provided in (Supplement 1) in the supplemental data in the online version of this article.

- Study Selection

**Figure 1:**
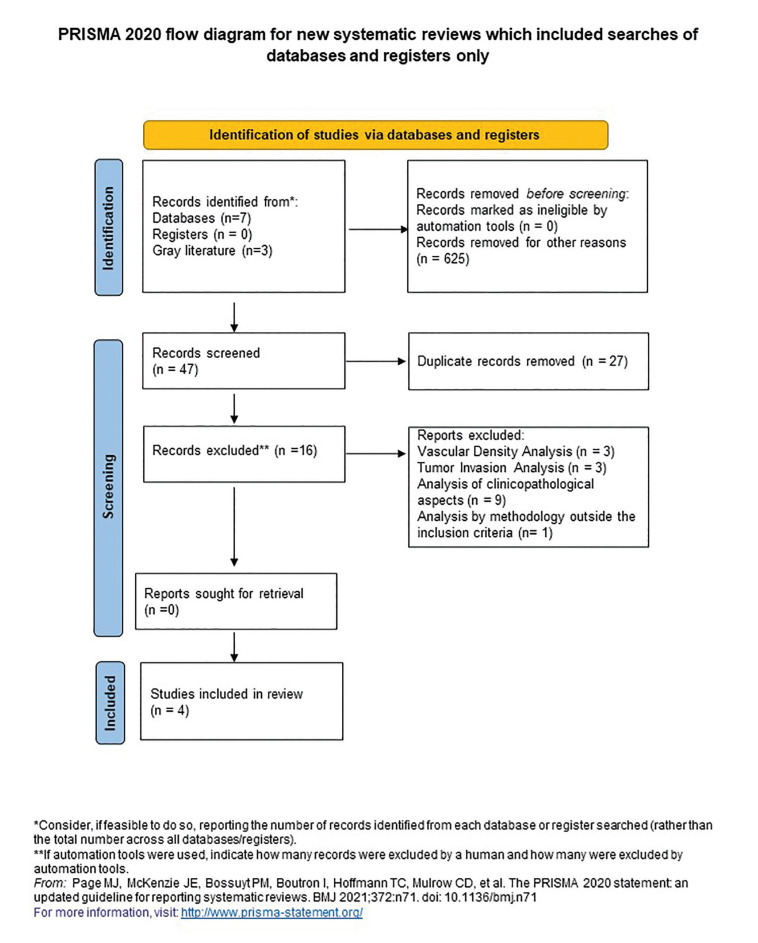
Flow diagram of the study identification, screening, and inclusion process. Adapted from Preferred Reporting Items for Systematic Reviews and Meta-Analyses.

According to a previous methodology outlined by de Paula *et al*. (10), study selection was completed in two phases. In the first phase, two reviewers (DSP and LVRL) searched the electronic databases adopted for the investigation. They independently reviewed the titles and abstracts of all electronic citations from databases related to the study using Rayyan®. Articles that did not meet the inclusion criteria were excluded. In the second phase, the preliminarily selected articles were reviewed according to the established inclusion criteria and the references were retrieved. The resulting list of included references was critically evaluated by a different reviewer (PGBS). Any disagreements were resolved by consensus among the three reviewers in the first or second phase of this search protocol. However, if the authors could not reach consensus, the other authors (PGBS and APNNA) were responsible for the final judgment. PGBS conducted the meta-analysis (Fig. 1).

- Data Collection Process

The data collection process included extracting information from the selected studies by one reviewer (LVRL), followed by a cross-check by a second reviewer (DSP). Two authors (DSP and LVRL) discussed any disagreements to reach consensus. If consensus was not reached, a third investigator (PGBS) made the final decision.

- Data Items

The selected studies were carefully evaluated, and the following specific variables were recorded: 1) sample size; 2) patient sex and age; 3) tumor staging; 4) IHC technique; 5) primary antibodies used; and 6) number of observers.

- Risk of Bias in Individual Studies

The risk of bias (RoB) was assessed by two independent reviewers (DSP and LVRL) who resolved any disagreements through discussions with a third author (PGBS). The methodological quality of the studies was determined using the Quality Assessment of Diagnostic Accuracy Studies-2 (QUADAS-2) tool (11). It consists of four main domains: domain 1 - Patient selection. Risk of bias: Can patient selection introduce a bias? Signaling question 1: Was there a consecutive or random sample of enrolled patients? Signaling question 2: Was the case-control design avoided? Signaling Question 3: Did the study avoid inappropriate exclusion? Applicability: Are there concerns that the included patients and settings do not match the review question? Domain 2 - Index Test. Risk of bias: might the conduct or interpretation of the index test introduce bias? Signaling question 1: Were the index test results interpreted without knowledge of reference standard results? Signaling question 2: If a threshold was used, was it prespecified? Applicability: Are there concerns that the index test, its conduct, or its interpretation differ from the review question? Domain 3 - Reference Standards Risk of Bias: Could the reference standard, its conduct, or its interpretation introduce bias? Signaling question 1: Is the reference standard likely to correctly classify the target condition? Flagging question 2: Were the reference standard results interpreted without knowledge of the index test results? Applicability: Are there concerns that the target condition, as defined by the reference standard, does not match this question? Domain 4 - Flow and Time Risk of Bias: Can patient flow introduce a bias? Signaling question 1: Was there an appropriate interval between the index test and the reference standard? Signaling question 2: Did all patients receive the same reference standard? Signaling question 3: Were all patients included in the analysis?

- Meta-Analysis

For the data synthesis, we extracted the false-positive, false-negative, true-positive, and true-positive cases for input into the Revman calculator and calculated the sensitivity, specificity, positive and negative predictive values, and accuracy of each. Thus, the data in the MedCalc® software were used to calculate combined diagnosis measures in the frequency meta-analysis of random effects. I² and Tau² were used to measure heterogeneity, and the 95% CI of the combined frequencies were used for the subgroup analysis.

- Evidence Quality

Evidence quality was assessed using the Grading of Recommendations, Assessment, Development, and Evaluation (GRADE) approach, which evaluates specific items based on estimates of the effect or reliability of association (9). The GRADE profiler summarizes evidence quality using GRADE Pro-GDT software (http://gdt. Guidelinedevelopment.org). Depending on the importance of some aspects (study design, RoB, consistency, frankness, heterogeneity, precision, publication bias, and others reported by studies included in the systematic review), evidence quality may be downgraded by one or two levels for each aspect.

## Results

- Characterization of OSCC Samples With Versus Without PNI and LVI

Four studies were included in the systematic review, and all were included and analyzed in the meta-analysis. All studies reported patient sex, with a total of 226 samples from female patients (of whom at least 42 had PNI) and 334 samples from male patients (of whom 54 were diagnosed with PNI) (Table 1). The ages of the patients evaluated varied widely. Kurtz *et al*. (4) analyzed patients aged 25 years, while Alkhadar *et al*. (12) verified OSCC in patients up to 105 years of age (Table 1).

**Table 1:**
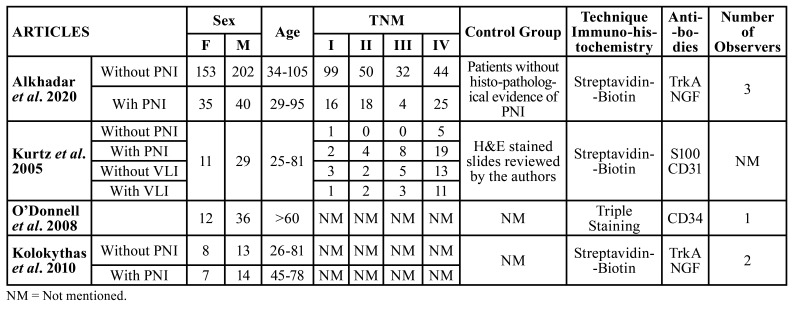
Demographic and clinical characteristics according to the presence and absence of perineural invasion and lymphovascular invasion.

Of the surveyed studies, only half mentioned tumor staging. Approximately 122 patients had stage I disease. Among them, 19 had PNI and LVI. Of the 76 patients with stage II disease, 24 were diagnosed with invasion of the analyzed spaces. A total of 52 patients had stage III disease, 15 of whom were diagnosed with PNI or LVI. In addition, 117 patients had stage IV disease, of whom 55 had PNI or LVI (Table 1).

Most of the studies analyzed used the streptavidin-biotin technique to perform IHC; only one used the triple staining method. Two used the same neural antibodies, nerve growth factor (NGF), and TrkA. Others analyzed PNI and LVI using antibodies against S100, CD31, and CD34. H&E and IHC slide analyses differed significantly, ranging from one to three pathologists among the included studies (Table 1).

- Meta-Analysis of Diagnostic Methods for PNI and LVI

Among the 295 pathology biopsies analyzed, approximately 76% (95% CI, 44.30–97.19%) of the samples evaluated by H&E detected PNI and LVI, while the IHC method was also able to identify them. The highest sensitivity was observed in the Alkhadar *et al*. (12) study, with 100% (95% CI, 94.13–100%) of PNI cases identified. There was significant inter-study heterogeneity (*p*<0.0001), with an inconsistency coefficient I² = 97.14% (95% CI, 95.54–98.16%) (Table 2).

The specificity of the four included studies was 42% (95% CI, 23.40–62.02%) for OSCC shown to have no PNI or LVI by HE. IHC analysis confirmed the negative diagnosis. The study by Kurtz *et al*. (4) evaluating LVI revealed the highest specificity of 82% (95% CI, 63.10–93.93%). Furthermore, it showed significant heterogeneity (*p*<0.0001) with an inconsistency coefficient of I² = 90.41% (95% CI, 81.86–94.93%) (Table 2).

Hence, the positive predictive value (PPV) was 61% (95% CI, 49.78–71.53%), with the study by Kurtz *et al*. (4) of PNI showing the highest value of 82% (95% CI, 67.22–92.66%). As with the previous analyses, significant heterogeneity was observed (*p*=0.0004) with the inconsistency coefficient I² = 77.91% (CI95% = 51.14 to 90.01%) (Table 2).

**Table 2:**
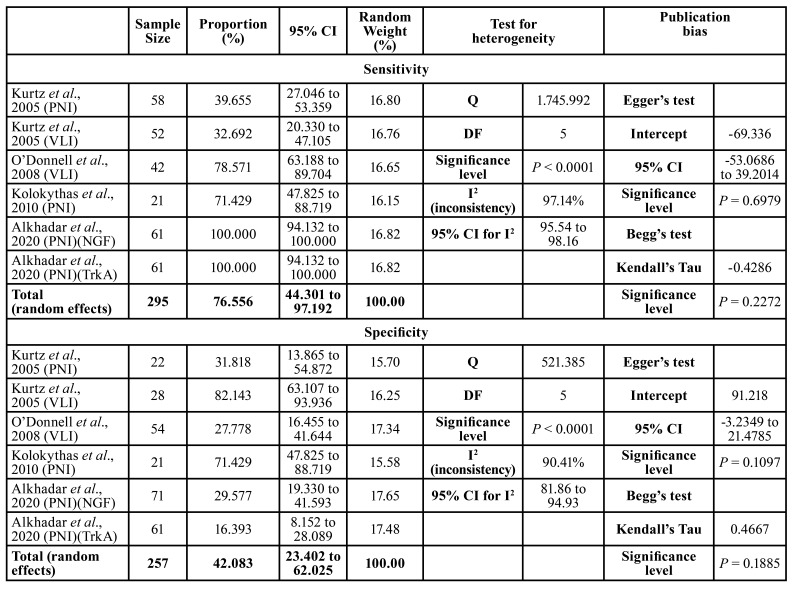
Frequency meta-analysis with calculations of sensitivity, specificity, positive and negative predictive values, and accuracy of immunomarkers used as predictors of perineural invasion or /lymphovascular invasion.

**Table 2 cont.**
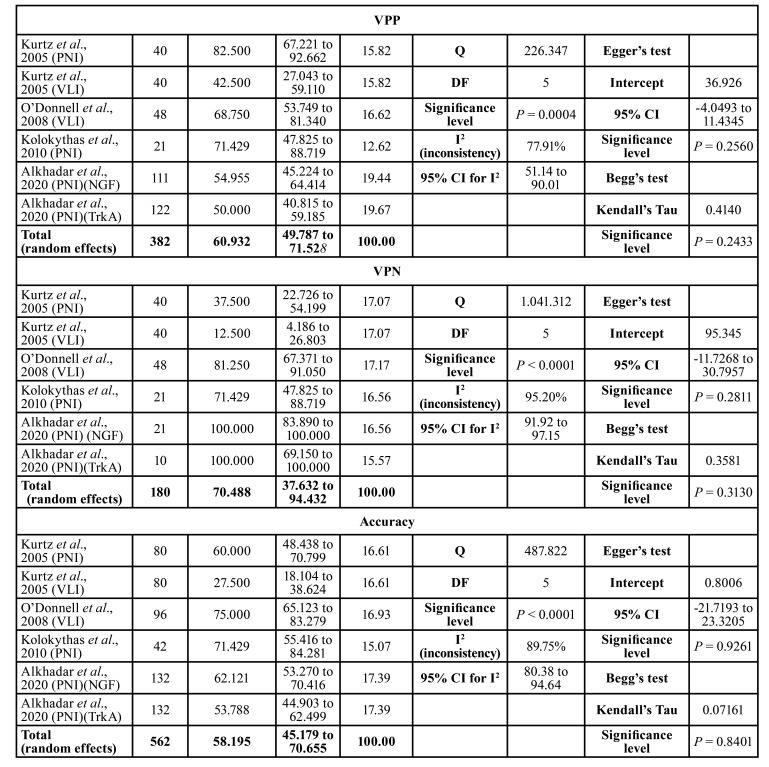
Frequency meta-analysis with calculations of sensitivity, specificity, positive and negative predictive values, and accuracy of immunomarkers used as predictors of perineural invasion or /lymphovascular invasion.

The negative predictive value revealed a total of 70% (95% CI, 37.63–94.43%). When analyzing perineural and lymphatic vessel invasion, Alkhadar *et al*. (12) obtained an NPV of 100% (95% CI, 83.89–100%) and pointed out significant heterogeneity (*p*<0.0001) with inconsistency coefficient of I² = 95.20% (95% CI, 91.92–97.15%).

Accuracy, on the other hand, was 58% (95% CI, 45.17–70.65%) and furthermore exhibited significant heterogeneity (*p*<0.0001) with an inconsistency coefficient of I² = 89.75% (95% CI, 80.38–94.64%) (Table 2).

- RoB Analysis

**Figure 2:**
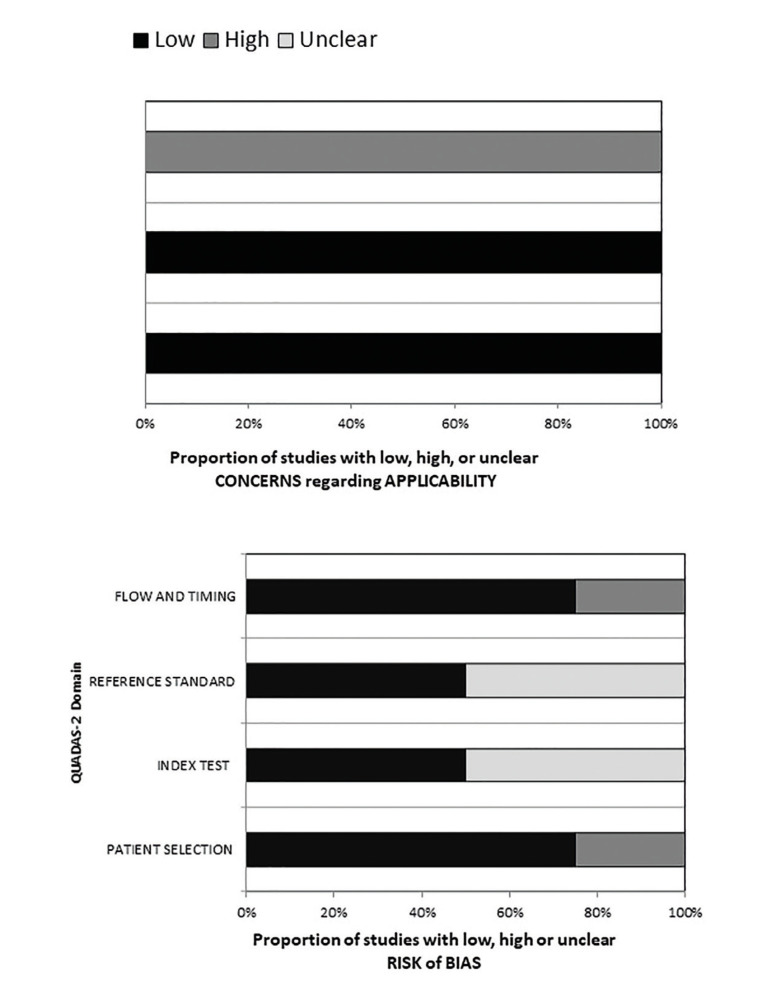
Risk of Bias Summary: Review the authors’ judgments on each risk item bias for each study included in the systematic review by the Quality Assessment of Diagnostic Accuracy Studies-2.

The four studies included and analyzed using the QUADAS-2 tool showed low risk. In domain one (patient selection), more than 70% of studies showed low risk, unlike domains two and three (index test and reference standard), for which 50% of the articles showed uncertain risk and the others showed low risk. In domain four (flow and timing), approximately 75% of the studies showed low risk, while the remainder showed high risk. In terms of applicability, domains one (patient selection) and two (index test) of 100% of the studies showed a low RoB; however, in domain three (reference standard), all analyzed articles exhibited a high risk of bias (Table 3 e Fig. 2).

**Table 3:**
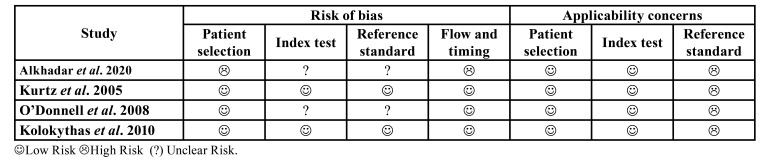
Risk of Bias Summary: Review the authors’ judgments on each risk item bias for each study included in the systematic review by the Quality Assessment of Diagnostic Accuracy Studies-2.

- Analysis of Evidence Certainty

The GRADE analysis showed a low evidence certainty. The lowest quality items were inconsistent and imprecise with severe scores, significantly reducing the evidence certainty (Table 4).

**Table 4:**
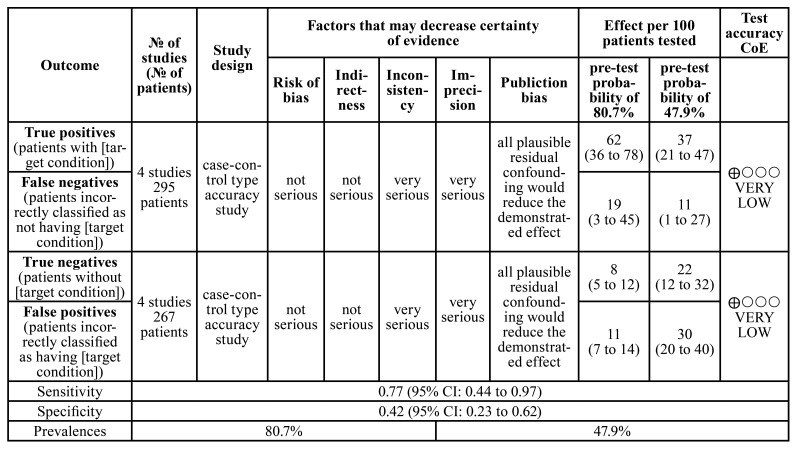
Grading of Recommendations, Assessment, Development, and Evaluation analysis of certain of evidence.

## Discussion

Surgical resection, the mainstay of treatment for OSCC, may be associated with adjuvant therapies following adverse histopathological features, such as close or involved surgical margins, PNI, LVI, and extracapsular dissemination (13). Since PNI and LVI are important components of the histopathological reporting of OSCC, they may be adverse prognostic indicators of local and regional recurrence, metastasis, and overall survival (14).

PNI is a parameter under consideration for providing information related to tumor aggressiveness. Although the Royal College of Pathologists of the United Kingdom and the College of American Pathologists noted the existence of PNI in histopathological analyses, its accuracy is considered controversial and subjective (15).

Some studies demonstrated that PNI is related to disease recurrence and the possibility of distant metastases; consequently, it reflects patient survival. The tumor cells that invade the perineural space are more aggressive, suggesting an increase in the tumor’s stage and grade (15,16). This corroborates our findings: among 117 patients with stage IV disease, 55 had PNI or LVI versus 122 patients with stage I disease, among whom only 19 had PNI and LVI.

LVI was included as a prognostic factor in the eighth edition of the AJCC Cancer Staging Manual. However, its relationship with the risk of recurrence and lymph node metastasis remains relatively unexplored. Studies have shown that LVI leads to a negative prognosis since it may be associated with metastasis (17). The presence of LVI indicates that a significant number of tumor cells enter the vascular compartment, which makes this feature one of the first steps for the potential development of metastasis (18).

In this sense, using diagnostic methods with greater specificity and sensitivity for PNI and LVI is of utmost importance in the application of the necessary adjuvant therapies and the increased survival of these patients (19). According to the meta-analysis data, the IHC technique can identify the cases evaluated by H&E and result in a false-negative result since the observed sensitivity of 76% was higher than the positive predictive value of 61%.

The use of IHC to stain nerves for diagnosing OSCC may be useful for detecting PNI (20). The reassessment of OSCC by IHC using anti-S100 increased the PNI detection rate from 30% to 82% (4). However, in the study by Barrett *et al*. (14), PNI was found in only five (8.3%) of the 60 OSCC initially reported negative cases. Shen *et al*. (21) exhibited an initial PNI detection rate of 22%, which increased to 39% after H&E re-evaluation and 51% after immunostaining with S100.

Kurtz *et al*. (4) reported vascular invasion in 30% of cases. After a slide review by the authors, 35% of the cases were interpreted as having vascular invasion. Immunolabeling with CD31 revealed vascular invasion in 42% of cases, including six false-positive and 11 false-negative cases of vascular invasion in the original reports. False-negative cases of LVI were also identified with IHC staining for CD34 (7).

PNI-positive OSCC samples expressed NGF and TrkA at a higher frequency than PNI-negative OSCC (12). Very similar findings from previous studies (21,22).

The discrepancies in the results reported in these studies may be partly attributed to the different sensitivities of detecting perineural and vascular invasion, techniques employed in IHC, and the number of observers. Among the four studies included in the systematic review and meta-analysis, three used the streptavidin-biotin technique (4,12,22), and only one applied triple staining (7).

Conventional IHC stains are useful, but there are significant limitations in the number of markers identified and localized per tissue section (23). However, triple staining can highlight and differentiate tumor presence between blood and lymphatic vessels, can be stored for long periods, is cost-effective, and does not require fluorescence. However, this is a more complex technique because color overlap and antibody cross-reaction can occur (7).

In addition to slides defined as controls, definitions of PNI and LVI used are another essential factor that can alter the sensitivity of the diagnosis and, consequently, the prevalence of the findings (20). Although some studies referenced and stressed the definitions of invasion for the analyzed spaces (4,12), others (7,22) did not clarify their criteria. In addition, the diagnostic criteria differ among pathologists, and there is less than moderate agreement in the evaluation of PNI in OSCC (5).

The analysis of retrospective studies in this meta-analysis has inherent limitations, such as variations in treatment approaches, underreporting of important information such as the definition of perineural and LVI employed, and the difference in the number of observers in each study. This is reflected in the low certainty of GRADE evidence observed, and further studies are vital for the findings of this systematic review to be applicable in the routine histopathological diagnosis of SCC of the mouth since perineural and lymphovascular vessel invasion are mechanisms of tumor dissemination and may represent a tumor eradication challenge.

## Conclusions

Our data demonstrated that using IHC stains to highlight tumor invasion into nerves and blood or lymphatic vessels could be effective in cases in which H&E analysis results are negative to decrease the prevalence of false-negative cases.
